# Fabrication and Tribological Performance of Self-Lubricating Porous Materials and Composites: A Review

**DOI:** 10.3390/ma17143448

**Published:** 2024-07-12

**Authors:** Ashish K. Kasar, Subin Antony Jose, Brian D’Souza, Pradeep L. Menezes

**Affiliations:** Department of Mechanical Engineering, University of Nevada, Reno, NV 89557, USA; ashishkasar91@gmail.com (A.K.K.); subinj@unr.edu (S.A.J.); brian39dsouza@gmail.com (B.D.)

**Keywords:** porosity, coefficient of friction, wear rate

## Abstract

Porous materials have recently attracted significant attention in the aerospace and biomedical fields for addressing issues related to friction and wear. Porous materials are beneficial in applications where continuous lubrication is not feasible or for components that operate under extreme conditions, such as high speeds, elevated temperatures, and heavy loads. The pores can serve as reservoirs for liquid lubricants, which are gradually released during the operation of the components. The tribological properties of these materials depend on their porosity, the lubricants used, and any additional additives incorporated into the porous materials. This review article provides insight into common fabrication techniques for porous materials and examines their tribological performance for all three classes of materials—polymers, metals, and ceramics. Additionally, it discusses design criteria for porous self-lubricating materials by highlighting the critical properties of both the substrate and lubricants.

## 1. Introduction

Porous materials are engineered with voids or open spaces within their structure to provide several distinct benefits. Porous materials can be classified into two types based on their porosity—open pores (through porosity), which can provide the passage of fluids, and closed pores (independent voids). The pores can be further classified based on their size. Pores 2 nm or smaller are called micropores, those ranging from 2 nm to 50 nm are known as mesopores, and pores larger than 50 nm are referred to as macropores [1]. Porous materials have a high surface area due to their internal structure, making them useful for various applications for all three classes of materials—polymer, metal, and ceramic. Porous polymers are often used as a drug delivery system, gas storage, sensing devices, and membranes of filtration [2,3]. Porous metals and alloys are frequently employed for their lightweight structural strength, catalytic capabilities, and roles in the filtration process and heat exchangers [4]. Porous ceramic materials find applications in filtration, separation processes, thermal insulation, and more [5]. Both porous metals and ceramic materials are extensively utilized in biomedical implants, serving as scaffolds for bone and tissue growth [6,7]. Porous materials offer superior thermal properties, including an increased convective heat transfer coefficient and higher thermal conductivity, relative to their base materials. It makes porous materials highly suitable for solar energy applications [8].

The application of porous materials in tribology is independent of the aforementioned application and has evolved over time. One notable early use of porous materials in tribology is the use of porous bronze bearing, which dates back to the early to mid-20th century by the automobile industry [9,10]. The voids present in the sintered bronze allow for the retention of lubricating oil and release of it to the surface during sliding, providing a self-lubricating capacity. The application of porous material in the field of tribology helps to overcome the limitations of conventional lubricating strategies that involve the continuous application of oil/grease on moving machinery. The continuous supply of lubricants is challenging in extreme conditions, especially in hard-to-reach places and when continuous lubrication is not possible. For such conditions, porous self-lubricating materials have gained a lot of attention in recent years to solve friction, wear, and lubrication problems [11,12]. These materials are capable of storing lubricants in their pores, which can help to reduce the friction of the components, hence making them useful in extreme conditions [13]. The lubricants stored in the pores can be released during sliding when a load is applied, and their effectiveness can be influenced by the temperature of the surrounding environment [14,15]. In addition, depending on the material–lubricant pair and pore size, the reabsorption of the lubricant can be realized through capillary action under static conditions. This enhances the recyclability of lubricants [16]. These self-lubricating porous materials find applications in equipment operating at high speeds, elevated temperatures, and heavy loads [17]. For example, Wang et al. [18]. demonstrated that polyimide as a retainer for a roller bearing failed in the first 1000 s of the test at 800 RPM under the axial load of 500 N, whereas a porous polyimide with filled oil did not show any sign of lubrication failure, even after 12 h of testing under the same testing conditions.

Engineering porous material for self-lubrication can be challenging. Different parameters need to be considered while designing a porous self-lubricating material. The first important factor is the type of material based on the application. In this review article, three classes of matrix materials are covered: (1) polymers, (2) metals, and (3) ceramics. Each class of self-lubricating porous materials has its applications and is specifically designed for certain environments and operating conditions. For example, ceramic substrates can have higher chemical and thermal resistance due to their structural stability compared with polymers or metals.

The pore size, pore concentration, and pore distribution of all the materials can be controlled by fabrication techniques, optimizing the type and concentration of additives and pore-forming agents. A wide range of fabrication methods, ranging from powder metallurgy to 3D printing, are discussed, with a focus on each method’s capability to control pore size and distribution. The matrix material plays a crucial role in impregnating and supplying lubricant in porous materials. The significance of considering surface energy is extensively discussed in the design criteria for self-lubricating materials. The tribological properties of various porous self-lubricating materials are discussed in terms of lubrication regimes and longevity.

## 2. Fabrication of Porous Materials

Porous polymeric materials are fabricated using various techniques, with some manufacturing processes being tailored to specific classes of porous materials. The initial step in the fabrication process involves selecting the matrix materials. Various types of materials are utilized as the matrix, chosen according to the specific application requirements. Some commonly used matrix materials for porous polymers are polyetheretherketone (PEEK) [19,20], polyphenylene sulfide (PPS) [21,22], and polyimide (PI) [23,24] due to their exceptional mechanical strength, chemical resistance, and high-temperature stability compared with other polymers.

**Figure 1:**
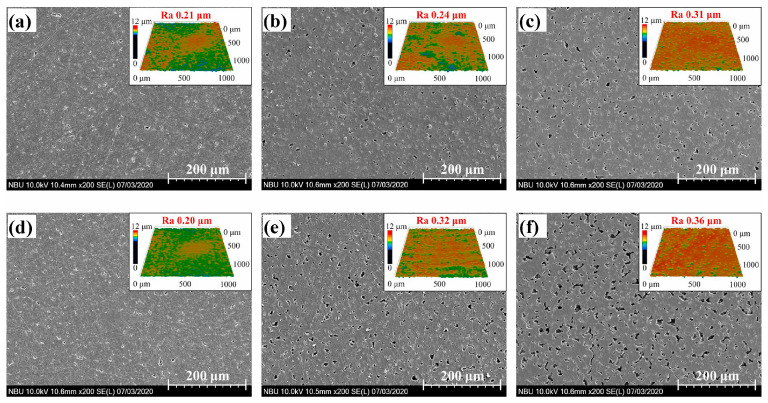
Surface micrographs of the polyimide substrate after laser scanning: (**a**) 5 W, 1 cycle, (**b**) 5 W, 10 times, (**c**) 5 W, 30 times, (**d**) 6 W, once, (**e**) 6 W, 10 times, and (**f**) 6 W, 30 times [25].

One of the reasons for pore formation in materials is the defects that arise during fabrication processes, such as the combination of compression and heat treatment. For example, polyimide porous substrates were prepared by compressing the material within a fixed volume to achieve a 1.1 g/cm^3^ density. Subsequently, the compressed substrate was heat treated at 350 °C for 1 h to promote densification [25]. This compression and heat treatment fabrication route often results in closed surface pores as the surface stays in contact with die/mold walls. For the infiltration of lubricants, post-processing methods are required to remove the top surface of closed pores, which can vary from simple processes such as grinding or polishing [23,26] to laser etching. For example, Ye et al. [25] generated different pore sizes on the polyimide substrate using different sets of laser power (5 and 6 W) and a different number of scanning cycles (1 to 30 times). The surface of the substrate resulted in different pore sizes, as shown in the Figure 1. However, though this study did not report pore size values, the surface roughness (R_a_) increased from 0.21 to 0.36, which indicates an increase in pore size.

Another technique for creating pores is through the addition of pore-forming agents such as sodium chloride (NaCl) [15,27]. Three-dimensional (3D) printing is another newer process that is used to fabricate porous polymers. Several techniques within the 3D printing process can produce porous polymers, such as designing pores in the print path [28,29], polymer matrices with sacrificial fillers [30,31], pores formed by solvent evaporation, and printing polymers with intrinsic porosity.

**Figure 2:**
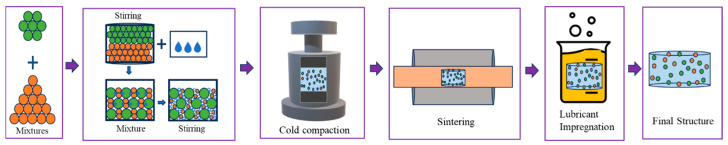
Schematic of a typical powder metallurgy method for self-lubricating composites starting from individual powder, mixing of powders, cold compaction followed by sintering to achieve porous material and liquid impregnation to achieve final structure of self-lubricating composite. The arrow shows the directionality in the process.

Metals and ceramics materials have similar fabrication techniques for porous materials that can carry lubricants. The most common technique is powder metallurgy, which uses powders that are pressed in a die or molds to produce green samples. Then, these green samples are heated to a suitable temperature, time, and environment to produce the desired part. The powder metallurgy process provides control over pores at both stages—pressing to produce a green sample—and also during sintering [32]. Apart from this, the addition of reinforcement particles can also limit the sintering and result in porous [33,34] materials. A typical powder metallurgy method shown in Figure 2 includes mixing, cold compaction, and sintering followed by lubrication impregnation. This method has been used in various studies. For example, porous metallic Cu-Sn-Ti composites [35] were reinforced with cubic boron nitride (CBN) and graphene nanoplatelets, where these powders were blended with carbamide particles using the ball milling technique for 30 min. Carbamide is a water-soluble organic compound, also known as urea, that is used as a pore-forming agent. These mixtures are subsequently pressed unidirectionally in a single-ended mold at 300 MPa for 30 s. To leach carbamide particles, these green samples were placed in distilled water at room temperature for 4 h. After drying, the samples were sintered at 880 °C with a holding time of 30 min under a high vacuum environment.

This inherent porosity is due to limited sintering that occurs uniformly by the formation of necks between powders. In addition to inherent pores during sintering, larger secondary pores can be obtained by space holders and gaseous blowing agents [36]. Space holders are typically the materials that can be burnt during sintering or washed away after sintering, e.g., NaCl [37], ammonium hydrogen bicarbonate [38], and polymeric materials [39]. These space holders are mixed with metal or ceramic powders before compaction, which are subsequently removed during or after sintering. Moreover, lower melting point metals can also be used as a space holder to generate higher porosity. For example, Mg has been used in Ti alloys to generate a porosity of up to 50–65%, where Mg evaporates during sintering without reacting with Ti [40]. Using a gaseous blowing agent, secondary large pores can be obtained. This technique is widely used to generate foam materials with a porosity of 90–95% for all three classes of materials: polymers [41,42], metals [43], and ceramics [44].

**Figure 3:**
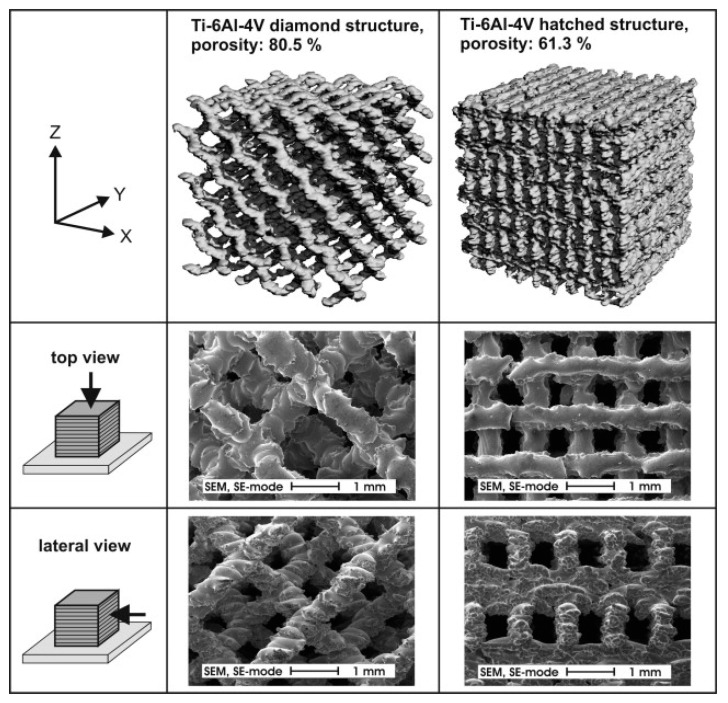
Porous diamond and hatched structure of Ti-6Al-4V [46].

The additive manufacturing (AM) of metals and ceramics provides the most attractive methods for fabricating porous materials, which is useful for creating complex functional materials while retaining good properties. The AM process is a layer-by-layer material deposition technique using an energy source such as an electron or laser beam that can yield a precisely controlled internal and external structure [45]. These porous structures are widely used in implants for bone–tissue engineering. One such porous Ti-6Al-4V alloy’s diamond and hatched structure as produced by a selective electron beam is shown in Figure 3 [46]. These porous Ti alloys exhibited maximum compressive strength in the range of 20–30 MPa for the diamond structure and 125–148 MPa for the hatched structure. These studies on additive manufacturing show that an optimal porous material can be prepared to control the volume porosity, corrosion resistance, and wear resistance [47,48]. The cold spray (CS) process is another coating/additive manufacturing technique capable of producing porous structures [49]. By adjusting the spraying parameters, the porosity level within the deposit can be controlled [50]. Wathanyu et al. [51] utilized the CS process to fabricate Ti porous coating on 316L stainless steel substrate by controlling the process gas temperature and pressure. However, the CS process is limited by material selection, as sufficient deformation of the spraying particles is required for effective bonding. The other methods for generating porosity in metals and ceramics are fugitive scaffolds of polymers [52], sol-gel processing [53], gel casting [54], etc.

## 3. Designing the Criteria and Lubrication Mechanism for Porous Self-Lubricating Composites

Reducing friction and wear are two very important objectives when designing porous self-lubricating composites. Friction is defined as the resistance to relative motion between two solid surfaces. The coefficient of friction (COF) is the ratio of the force of friction to the normal force applied, and it is a dimensionless number. Wear is defined as the progressive loss of material during sliding between two bodies. The lubrication mechanism of the porous composites can vary depending on various factors such as the type of material used as the matrix, the fabrication process, the pore-forming agents, and the lubricants. Firstly, the design criteria and lubrication mechanism are discussed in the following section.

**Figure 4:**
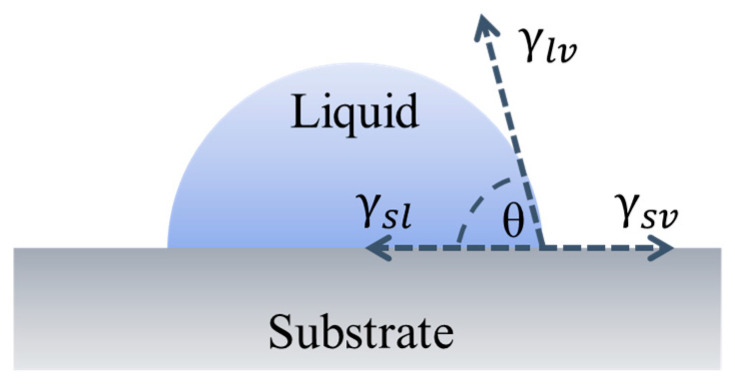
Schematic of a liquid droplet on the substrate with surface energy components.

The first step in the development of a porous lubricating material is the impregnation and storage of lubricants. The impregnation of the liquid lubricants depends on both the substrate and liquid lubricant properties. The critical properties of the substrate are the pore size and surface energy, whereas the critical parameters for the liquid lubricants are the surface tension (γ) and viscosity (η). On a smooth substrate, the spreading of liquid as measured in terms of the contact angle can be calculated by Young’s equation [55] and schematically shown in Figure 4.

(1) $$\cos\theta =\frac{\gamma_{sv}-\gamma_{sl}}{\gamma_{lv}}$$

where *θ* is the contact angle between substrate and liquid and $\gamma_{sv},\;\gamma_{sl},\;\gamma_{lv}$ are the surface energy of the solid, interfacial surface tension, and surface tension of the liquid, respectively. For the liquid impregnation rate, Waldner and Hirn [56] reported that the Lucas–Washburn (LW) equation [57] can be used to represent the flow of liquid under the assumption of circular pores and capillary pressure as a driving force. The LW equation is given as

(2) $$\frac{dh}{dt}=\frac{r\gamma_{lv}\cos\theta }{4\eta h}$$

where *r* and *h* are the pore radius and impregnation depth, respectively. By replacing the contact angle term from Equations (1) and (2), impregnation depth can be estimated using surface energy values. However, the measurement of surface energies ($\gamma_{sv}\;and\;\gamma_{sl}$) is difficult to perform. Therefore, surface energy modeling approaches can be used. The most widely known surface energy estimation approaches are Owens, Wendt, Rabel, and Kaelble (OWRK) [58], van Oss, Good, Chaudhury (vOGC) [59], and Wu [60]. The OWRK is based on the dispersive and polar components and vOGC and Wu are based on the acid–base components. These approaches are widely used for surface energy measurement using suitable liquids. Waldner and Hirn [56] developed this approach by combining the Young equation, LW equation, and these surface energy estimations to test the penetration depth for mixed liquids of water, glycerol, hexanediol, and naphthol blue black dye on a porous media, which yielded a higher accuracy (R^2^ > 0.75) concerning experimental results. This combined approach of liquid flow and surface energy suggests that the most important parameters to design a porous self-lubricating system are the viscosity for the liquid lubricant, whereas it should be surface energy and pore size for the substrate material. The most technique for liquid impregnation inside the porous substrate is vacuum impregnation at higher temperature. The application of vacuum helps in speeding the impregnation process by taking out the entrapped air inside the voids, whereas higher temperature reduces the viscosity of the liquid lubricants to flow inside the voids.

Once the liquid lubricant is impregnated in the porous substrate, the lubrication mechanism during sliding against a counterbody is explained by the sweating mechanism. In this mechanism, as the material is subjected to friction during sliding, it releases the liquid stored in the pores under capillary force [61,62]. The coupled effect of load and sliding increases temperature, which assists the liquid lubricant to flow and form a layer on the surface of the material, preventing direct contact between the two materials. The increase in temperature at the asperity level (flash temperature) and bulk temperature at the surface can be estimated by the model developed by Ashby et al. [63]. Also, the liquid lubricant is restored in the pores when the material is not in operating conditions [64]. In this sweating mechanism, the release of liquid lubricants under capillary force is not the only factor; the deformation of the lubricant-carrying substrate is also a contributing factor. Zhan et al. [65] considered the porous lubricating system as a solid–liquid biphase model and analyzed the system numerically under different loading conditions to estimate the amount of stress on the solid (substrate) and fluid pressure (lubricant). The deformation of the substrate causes an increase in fluid pressure that is the driving force for the seepage of lubricants and spread over the sliding interface.

**Figure 5:**
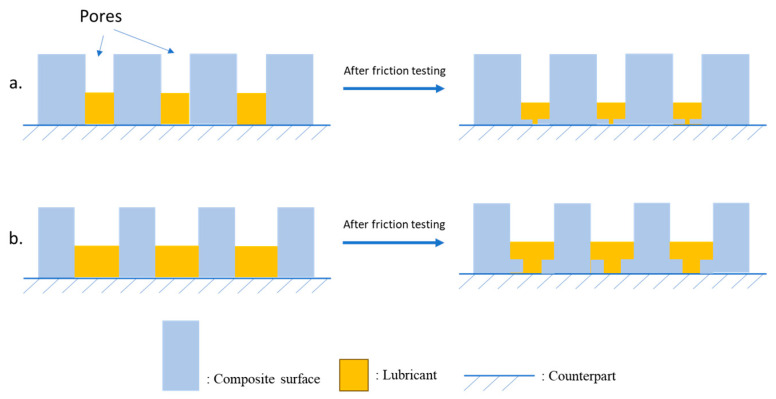
(**a**) Higher relative density substrates have closed pores that hold less lubrication, providing poor lubrication performance. (**b**) Lower relative density substrates are more prone to wear, which can break weak joints and close pores, leading to less lubrication.

Pores play an important role in lubrication mechanisms for porous materials. Pore generation relates to the fabrication of the porous substrate. Controlling pores is related to the relative density of the matrix materials. Relative density is defined as the ratio of the measured porous substrate density to its theoretical density. In general, substrates with higher relative density (>90%) will lead to closed pores that are not connected, which limits the lubrication carrying capacity of the materials, and starved lubrication yields poor lubrication performance. On the other hand, lower density to achieve connecting open pores will have poor mechanical strength due to weak particle-to-particle strength. During sliding, the weak joints can break and cause higher wear. Additionally, the increase in wear may lead to the closing of pores or a reduction in pore size that limits lubrication and increases friction, as shown in Figure 5. Therefore, optimized pore conditions or density are required to achieve consistent lubrication performance in the impregnated porous substrate materials. Close pores on the substrate can limit the lubrication carrying capacity of the materials, whereas higher pores reduce the overall strength of materials. Therefore, the pore size is a critical design parameter for self-lubricating porous material.

### 3.1. Polymer Matrix Porous Composites

In polymers, there are three principal classes—thermoplastics, thermosets, and elastomers. Among these three, thermoplastics and thermosets are often known as polymers and are most suitable for porous matrix material due to their higher stiffness [66]. The Young’s modulus of thermosets and thermoplastics is typically one to two orders of magnitude higher than those of elastomers, which makes them ideal candidates because contact pressure during sliding can be in the order of 1 GPa [67]. Meanwhile, elastomers are widely suitable, wherein structure rigidity is not required due to their extremely high flexibility.

In a study conducted by Wang et al. [61], porous PEEK composites were filled with ionic liquid (1-butyl-3-methylimidazolium hexafluorophosphate), and tribological tests were performed. The study found that PEEK composites with nano/micro porous structures (23.6% porosity) impregnated with ionic liquid showed excellent tribological properties compared with pure PEEK composites. The impregnation was carried out at 80 °C for 2 h under vacuum. The tribological test was conducted using a ring-on-ring setup at 250 N and 0.69 m/s against a 1045 steel ring. The ionic liquid-impregnated PEEK yielded COF = 0.05, which is 65% less than dry PEEK, and a wear rate of 2.0 × 10^−14^ m^3^/Nm, which is 10^3^ times less than that of dry PEEK. A similar study by Liu et al. [62] showed that the micro/nano porous carbon fiber/polytetrafluoroethylene/PEEK composites impregnated with 1-hexyl-3-methylimidazolium tetrafluoroborate ionic liquid yielded the minimum COF of 0.0197 and wear rate of 4.14 × 10^−15^ m^3^/Nm. For comparison, the COF of PEEK against 100Cr6 Stainless steel under dry sliding conditions is ~0.6, and the wear rate is ~3.0 × 10^−6^ m^3^/Nm, which is much higher than that of ionic liquid-impregnated PEEK. The addition of solid lubricants such as nano-to-micron WS_2_ and MoS_2_ to PEEK can help in reducing the COF to 0.4 and wear rate by ~50%, which are still higher [26]. These enormous differences in the COF and wear rate of dry PEEK versus liquid-impregnated PEEK suggest that impregnated porous materials are the solution to increase the life of the machine components.

In the porous self-lubricating composites, lubrication regimes can also be altered by using different porosities. Wang et al. [23] synthesized porous polyimide (PI) composites with different porosities ranging from 16 to 20%, which assisted in retaining different oil (poly-α-olefin-PAO4) content. The tribological tests were conducted using a pin-on-disk setup at room temperature under loads of 100, 200, and 400 N with increasing sliding velocity from 0.025 to 1.875 m/s to understand lubrication regimes. The results were also compared with polyimide without any pores (PI0) being tested in fully flooded conditions (oil bath). For PI0, the friction coefficient increased with an increase in load, whereas the friction coefficient decreased for PI13 (polyimide with 13% oil content and 17.28% porosity) with the increase in load. The difference is more significant between mixed and boundary lubrication regimes. The higher load flushes away the oil and increases solid-to-solid contacts for PI0. In the case of PI13, the higher load caused the release of oil from the pores to the interacting surface and decreased friction. It is also important to notice that the electrohydrodynamic lubrication region occurred earlier under a higher load for PI13 due to the greater release of oil from pores. The other tribological studies on polyimide porous material are listed in Table 1.

**Figure 6:**
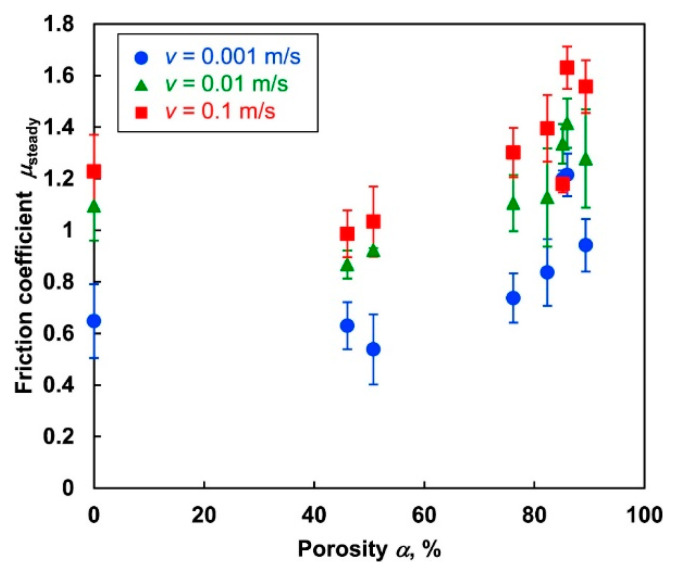
Effect of porosity on the mean value of the COF for different sliding velocities [68].

For porous elastomers, limited tribological studies have been carried out. In a study performed by Yamaguchi et al. [68], the authors investigated the dry sliding friction behavior of non-porous and porous ethylene-vinyl acetate (EVA) blocks with various porosity (0 to 90%) values against a stainless steel plate. Figure 6 depicts the results that showed that the highly porous EVA block provided larger contact areas due to the elastic collapse at the interior part of the block, which resulted in a high COF. In contrast, low porosity reduced the COF through a reduction in the contact area resulting from an increase in surface pores. Additionally, a decrease in velocity increased the COF, which was due to increase in the length of the apparent contact area at a lower sliding velocity. These results suggest that soft, porous polymers can be utilized as light high-friction materials, such as lightweight shoe sole materials with high cushioning and high grip, under dry conditions.

As seen from many studies performed, porosity is inversely related to the tribological properties of a composite [32,69]. Increasing porosity decreases the hardness of the composites, which leads to a decrease in the friction and wear resistance of the materials. Hence, the material needs to have an optimum amount of porosity that does not significantly reduce the mechanical properties of the material.

Lubricants are added to porous composites to improve their friction and wear resistance. The type of lubricants incorporated in the pores can vary from solid to liquid lubricants. The latter is commonly used in porous materials as lubricants can be effectively stored in the pores. Solid lubricants are used in conditions where liquid lubricants may be lost during working conditions, such as gear and chain lubrication and reciprocating motion. In these cases, solid lubricants are helpful as they can provide continuous lubrication and there is no loss of lubricants, thereby preventing fretting corrosion [70].

### 3.2. Porous Metal and Ceramic Matrix Self-Lubricating Composites

**Figure 7:**
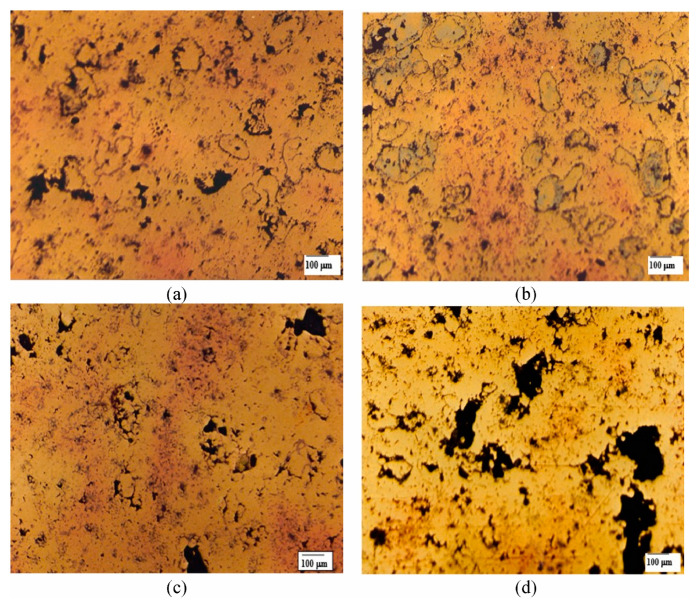
Microstructure of Cu-Sn-Zn composite with graphite at (**a**) 0.5, (**b**) 1.0, (**c**) 1.5 and (**d**) 2.0 wt.% [75].

Only a few studies have been carried out on porous metal and ceramic matrices infiltrated with lubricating agents. Among porous metal matrix materials, the most famous and widely utilized is porous bearing material, which has been in use since 1925 [71]. A review article by Kumar [72] published in 1980 discusses the various porous metal bearings and their applications. The porous aluminum bearing was high in demand due to its lower cost and high thermal conductivity. However, poor galling behavior limits aluminum bearings. Later on, sintered bronze and iron bearings (porous) were used, which showed a higher eccentricity ratio than solid bearings (non-porous). The eccentricity ratio of a bearing is defined as the ratio of eccentricity and radial clearance. It is also important to notice that the oil for such bearings under a high load-carrying capacity should have a higher oxidation resistance. The commonly used oil for porous materials was silicon oil. Moreover, oxidation inhibitors can also be added to enhance the oil life in the porous material. The amount of porosity is the key factor in metals and ceramic substrates as well. Guo et al. [73] utilized the powder metallurgy method to fabricate Fe-Cu-Ni-Sn graphite porous substrate sintered at 1080 °C with a 40% porosity and 182 μm surface pore size. The authors provided a secondary treatment of rolling at 900 °C followed by another sintering at 1080 °C to decrease the porosity to ~30% and pore size to 158 μm. It was observed that the secondary treatment was beneficial in reducing the COF from ~0.95 (without secondary treatment) to 0.55 during the pin-on-disk test. The wear was measured in terms of weight loss, which also reduced from 6.5 mg to 2.75 mg by secondary treatment. The improvement in the COF and wear performance is mainly due to the reduction in porosity and pore size by the secondary treatment. Higher porosity and larger pore size can decrease the substrate strength and yield higher wear. The resulting wear debris can block the pores and affect the continuous supply of lubricants. Moreover, the addition of graphite was also helpful in reducing the COF and wear with lubricating oil, which has also been shown by other porous lubricating metallic systems such as Cu-Sn [74], Ni [17], etc. The stored lubricating oil and graphite not only reduce the friction but also protect the oxidation of the substrate and form a carbon-based tribofilm. A typical microstructural of Cu-Sn-Zn-graphite composite for bearing material is shown in Figure 7, which shows increase in pore size from 25 to 37% due to the addition of graphite ranging from 0.5 to 2.0 wt.%.

While copper-based materials have been studied in the majority of papers as the self-lubricating porous substrate, austenitic stainless steel has also demonstrated similar benefits. Martin et al. [76] prepared 316 SS porous substrates by the powder metallurgy of different % pores by selecting the different initial powder sizes. The particle size < 150 μm yielded a 7.6% porosity, whereas the larger particle size (500–750 μm) provided a 28.8% porosity. The prepared samples were vacuum-impregnated at room temperature for 12 h with 0.5% aqueous solution of carboxymethyl cellulose sodium salt medium viscosity as a lubricant for biomedical applications, followed by tribological testing. The scar depth of the samples with 28.8% porosity remained consistent at ~0.05 mm during 200 m of sliding distance, whereas the scar depth continuously increased for the sample with a 7.6% porosity, with a final value of 0.2 mm at the end of the test, which was four times higher. The COF values also showed a similar trend with respect to porosity. It was found that the pores on the samples with lower porosity were closed after the initial sliding distance (<50 m) that restricted the lubricant supply.

Among ceramics, the widely used ceramic material is Al_2_O_3_, which has also been utilized for self-lubricating purposes. Rowthu and Hoffman [77] studied perfluoropolyether-impregnated alumina matrix materials where porous alumina matrix was prepared by slip casting followed by pre-sintering at 600 °C to remove the binding materials. The pre-sintered alumina matrix showed a relative density of ~59%, which was further sintered at different temperatures from 1150 to 1500 °C to achieve different levels of density, or in other words, different porosity. The porous alumina matrix substrates were impregnated with perfluoropolyether oil by submerging at 150 °C for 2 h. The authors observed that the composites with 90% relative density yielded a lower COF of ~0.13 at a 220 N load. The composites with lesser density had slightly higher COF values because of the weak particle-to-particle strength that caused higher wear and subsequently higher COF values. Meanwhile, the composites denser than 90% had closed pores that yielded higher COF values due to the absence of a continuous supply of lubricants. Based on Al_2_O_3_ ceramic, Kasar et al. [78] developed an Al_2_O_3_^−^ 20 wt.% B_2_O_3_ ceramic composite that generates inherent porosity due to the formation of the aluminum borate phase during pressureless sintering. The composite was impregnated with phosphonium-based ionic liquids under vacuum at 60 °C for 30 min. The authors suggested that the high-temperature impregnation was needed due to the high room temperature viscosity of the phosphonium ionic liquids, which also suggests the importance of viscosity during impregnation, as discussed in Section 3. After impregnation, pin-on-disc sliding tests were performed at four different temperatures from 5 to 100 °C and also at three different sliding velocities. At lower temperatures (5 °C) and lower sliding velocity (2 mm/s), slightly higher friction was observed compared with room temperature. This study suggests the effect of the increase in flash and bulk temperature at sliding surfaces that assist in ionic liquid supply at sliding interfaces. In addition, the suitability of ionic liquid for high-temperature applications was also discussed due to thermal stability.

Pores in materials typically deteriorate their mechanical properties, which is undesirable and can cause a decrease in friction and wear resistance. To improve these mechanical properties, additives are often used during the fabrication of the materials [79]. Like the pore-forming agents, the concentration of strengthening agents must also be optimized. Adding a high concentration of strengthening agents can have an adverse effect on the mechanical and tribological properties of the materials.

**Figure 8:**
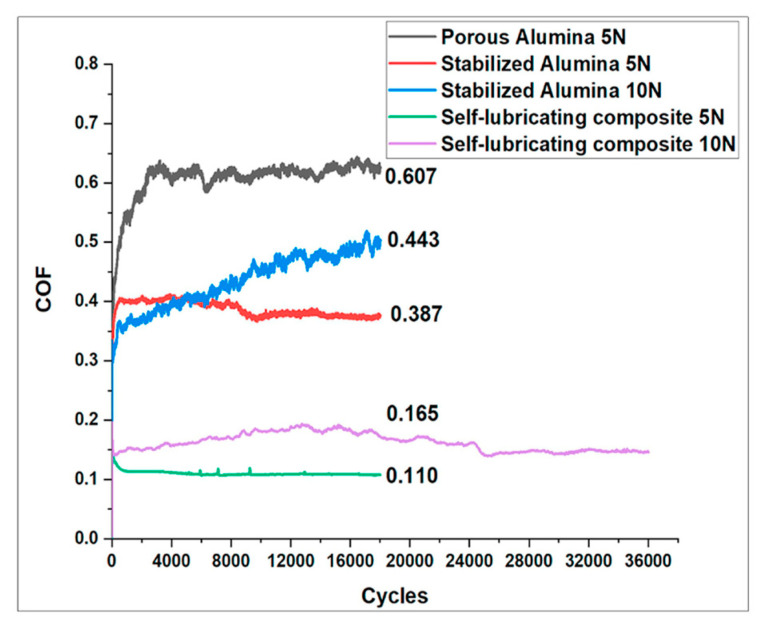
COF values for different alumina specimens under 5 N and 10 N loads [70].

Unlike liquid lubricants, a limited study has demonstrated the use of solid lubricants in porous materials to provide continuous lubrication. In a study performed by Salam et al. [70], inorganic hollow fullerene-like (IF) MoS_2_ was used as a solid lubricant in porous alumina substrates, and the tribological properties were studied. Three different specimens were tested: (1) porous alumina, (2) alumina stabilized with acrylic resins, and (3) hollow IF-MoS_2_ impregnated in alumina. Ball-on-disk reciprocating tests were performed using a zirconia (ZrO_2_) ball at 5 and 10 N loads for 18,000 cycles, and friction and wear results were compared between the samples. Figure 8 shows the COF results for the tribological tests. It can be concluded that the hollow IF-MoS_2_ specimen showed the least COF compared with the other two samples. The stabilization of alumina with acrylic resin helped to bring down the COF but could not maintain the low COF for an extended period. However, adding hollow IF-MoS_2_ significantly reduced the COF of the specimen and lasted for 36,000 cycles at 10 N load.

There are multiple lubrication mechanisms for the hollow IF-MoS_2_ particles. Their spherical shape acts as rollers that produce a rolling effect and reduce COF [70,80]. They also sit in between surface peaks and valleys, which help dissipate the energy produced by friction and wear during sliding. The hollow IF-MoS_2_ is not destroyed immediately; rather, it slowly deteriorates, which is helpful in the long run.

**Figure 9:**
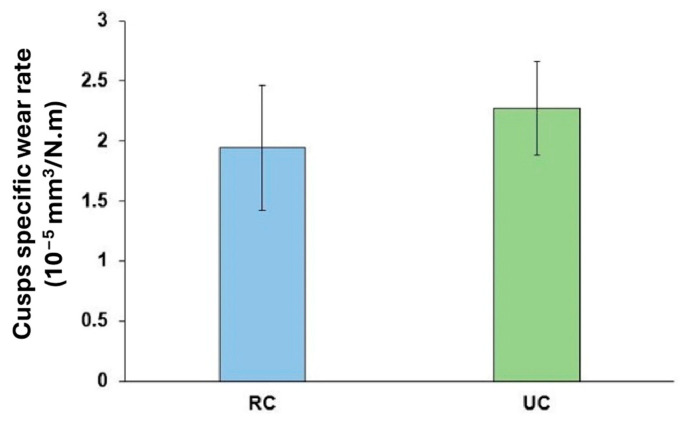
Specific wear rates of the dental cusps tested against 3D-printed (RC) and unidirectionally compressed (UC) plates [81].

A study conducted by Branco et al. [81] evaluated and compared the tribological properties of human teeth against zirconia dental prosthetics produced by robocasting (RC), a 3D printing technique with prosthetics produced by traditional unidirectional compression (UC). The RC samples were 3D-printed using a ceramic paste of 3 mol% Yttria partially stabilized zirconia at room temperature using a 0.4 mm diameter nozzle at 60 mm/s with a layer height of 0.2 mm. Meanwhile, the UC samples were produced under a load of 70 MPa for 10 s. Both the samples were sintered at 1500 °C for 2 h. The results showed that the RC samples had a higher surface roughness, lower hardness, and fracture toughness compared with the UC samples. These properties were attributed to the higher porosity of 3D-printed samples. Dental cusps were tested against both pieces to simulate chewing conditions in the presence of artificial saliva, which showed a similar specific wear rate as that shown in Figure 9. Both the dental zirconia pieces underwent mild abrasive wear, fatigue, and delamination. 3D printing techniques can fabricate products with complex geometry with identical tribological properties as the conventional method. Additionally, the higher roughness of RC samples due to porosity did not negatively affect the enamel wear. The pores could be advantageous for storing dental wear debris and avoiding sharp edges.

## 4. Conclusions

Porous materials can be used for various applications and have been gaining more attention lately. Porous materials are used to solve friction, wear, and lubrication problems. The pores in these materials are utilized to store liquid lubricants, which come out during sliding and return to the pores after operation. Porous materials can be classified into many different categories depending on the pore size, type of materials used, and location of pores. In this review, porous materials are categorized by the type of materials used and have three categories: porous polymers, metals, and ceramics. The three different types of porous materials have different properties, and depending on the application, a certain type of material can be selected. Porous materials are fabricated by many different methods, such as sintering, molding, casting, selective laser melting, and 3D printing. Depending on the application, different materials and processing techniques are used to fabricate porous materials. Porous materials usually have lower mechanical strength. Hence, the fabrication process also involves the incorporation of strengthening additives. All of these additives have an impact on the tribological properties of the materials. The design criteria are also discussed, which suggests that the optimization of pore size is needed based on the fundamental properties of lubricants, surfaces, and contact pressure. The observation of the elastohydrodynamic lubrication regime confirms that the porous self-lubricating composites are viable routes for achieving lower friction for the difficult-to-reach components. This review article also highlights that the type of liquid lubricants is independent of the matrix materials. In different porous polymer substrates, all of the different types of liquid lubricants such as ionic liquid, lithium grease, diesel oil, etc., have been successfully used to achieve superior tribological properties. The studies discussed in this article suggest the selection of the liquid lubricant is mainly based on the requirements, e.g., viscous ionic liquid with high temperature stability can be utilized in porous substrate where viscous lubricant is required at a high-temperature application. In addition to the technical advantages of porous self-lubricating components, the ability of the porous material to hold back the lubricants while not in motion provides sustainability and avoids the excess use of lubricants.

## Figures and Tables

**Table 1:** Tribological properties of porous materials impregnated with lubricants.

Matrix	Lubricant and Impregnation Condition	Testing Condition	% COF and Wear	Ref.
Polyimide—20% porosity	Silicone oil (32 h at room temperature in vacuum)	Reciprocating sliding test against GCr15 steel ball at 20 N	COF = 0.03 (~10 times lesser than that of dry non-porous polyimide)	[11]
Polyphenylene sulfide (PPS) + PTFE with TiO_2_ whisker	Lithium-based grease (2 h at 120 °C in vacuum)		Wear resistance improved by 6.45 × 10^3^ times compared with pure PPS	[21]
PPS with 1% zeolite (20% porosity)	Lithium grease (2 h at 120 °C in vacuum)	Ring-on-ring test at 150 N with 1.4 m/s	COF = 0.024 and 1.79 × 10^−16^ m^3^/Nm (COF and wear were 90% and 4.67 × 10^4^ lesser compared with dry PPS, respectively)	[15]
Polyimide (PPI) with 33.5% porosity	Diesel engine oil (12 h at room temperature in vacuum)	Ring-on-disk setup at 100 N and 0.22 m/s against stainless steel ring	COF = 0.1 (70% lesser than dry porous PI)	[18]
PEEK (16.8% porosity)	Ionic liquid: 1-butyl-3-methylimidazolium hexafluorophosphate (2 h at 80 °C in vacuum)	Ring-on-ring setup at 250 N and 0.69 m/s against 1045 steel ring	COF = 0.05 and wear rate 2.0 × 10^−14^ m^3^/Nm (COF and wear rate were 65% and 10^3^ and were lower)	[61]
PPI (17.48%)	poly-α-olefin oil (20 h at 100 °C in vacuum)	Ball-on-disk (GCr15 steel ball) at 10 N and 0.2 m/s	PPI sample with the smallest pore diameter (1.48 μm) yielded the lowest COF ~0.035	[25]

## Data Availability

No new data were created.
